# Temporal dynamics of the twinkle-goes illusion and its relationship to neural theta oscillations

**DOI:** 10.1007/s00221-025-07187-5

**Published:** 2025-11-14

**Authors:** Ryohei Nakayama, Kaoru Amano, Ikuya Murakami

**Affiliations:** 1 Department of Psychology, The University of Tokyo, 7-3-1 Hongo, Bunkyo-ku, Tokyo, 113-0033 Japan; 2 Graduate School of Information Science and Technology, The University of Tokyo, 7-3-1 Hongo, Bunkyo-ku, Tokyo, 113-8656 Japan

**Keywords:** Twinkle-goes illusion, Positional prediction overshoot, EEG, Theta phase

## Abstract

Visual motion signals are useful in predicting the future, and can affect the detectability and phenomenology of vision in various ways. Recent research has demonstrated that the disappearance position of a moving object is perceived as shifted in the direction of motion when the background consists of dynamic noise. This “twinkle-goes” illusion is thought to arise from a positional prediction overshoot that occurs because dynamic noise delays the accumulation of sensory evidence needed to register the disappearance. In Experiment 1, we examined the temporal dynamics of this illusion by measuring the illusory position shift using a probe at various positions along the motion trajectory and at different time points after the object’s physical vanishing. The illusory position shift was nearly zero at the moment the moving object vanished, and subsequently gradually increased as a function of time up to ~ 120 ms after vanishing. In Experiment 2, motivated by prior reports of rhythmic fluctuations in both behavior and neural activity, we investigated whether neural theta oscillations were involved in the illusion. We found that the size of the illusory position shift correlated with the theta phase before vanishing. Taken together, these results suggest that the positional prediction of a moving object is slow-paced and rhythmically updated in synchrony with theta oscillations.

**Supplementary Information:**

The online version contains supplementary material available at 10.1007/s00221-025-07187-5.

## Introduction

Visual motion signals contain the direction and speed information for predicting future positions of moving objects. Such positional predictions (i.e., internal estimates of where an object is heading based on its current trajectory) are not only useful in keeping track of object trajectories, but also helpful for other visual processes. The covert and simultaneous tracking of multiple moving objects (Cavanagh 1992; Pylyshyn and Storm 1988) is easier when they move linearly than when they exhibit unpredictable zigzag motions (Howe and Holcombe 2012; Luu and Howe 2015). Furthermore, one study demonstrated that the detectability of a grating presented at the leading edge of another moving grating was enhanced when these gratings were in phase (Roach et al. 2011).

**Fig. 1:**
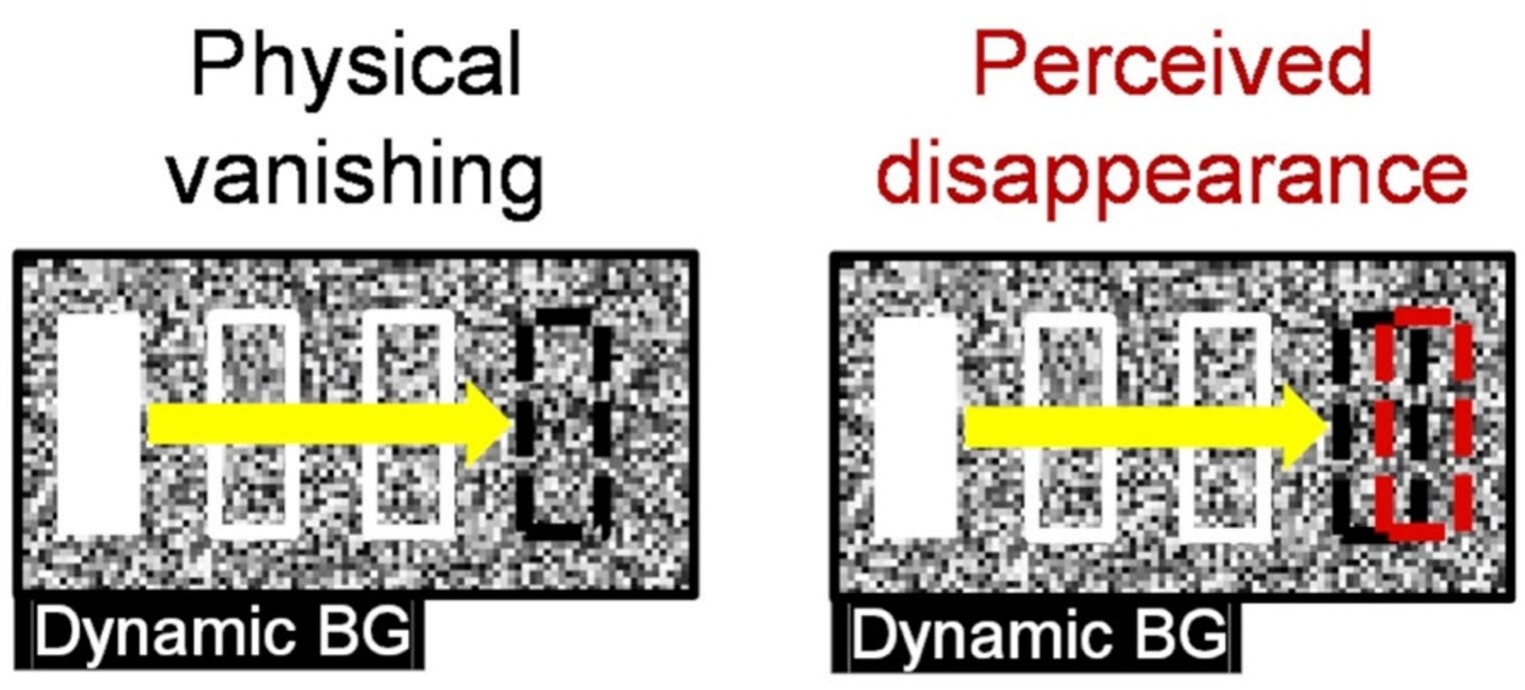
Illustration of the twinkle-goes illusion. The left panel shows a moving object (white bar) as it moves rightward (illustrated here by the yellow arrow and a series of white rectangles), with its physical vanishing position illustrated by a black dashed rectangle. The right panel shows the moving object’s perceived disappearance position, illustrated by a red dashed rectangle, which appears spatially shifted from the physical vanishing position in the pre-vanishing motion direction. The dynamic-noise background may mask the offset transient, enabling this perceptual overshoot to occur

Nakayama and Holcombe (2021) previously showed that the disappearance position of a moving object is perceived as shifted in the direction of motion with a dynamic-noise background (Fig. 1), demonstrating a dissociation between physical vanishing position and perceived disappearance position (hereafter, “vanish/vanishing“ denotes the physical deletion from the screen, while “disappear/disappearance“ denotes ceasing to exist in one’s conscious awareness). Importantly, disappearance detection is also slowed when the background is dynamic rather than static noise (Nakayama and Holcombe 2021), consistent with the notion that dynamic noise masks the offset transient that normally acts as a “reset signal”. Such masking both delays the registration of disappearance and allows positional prediction to continue, producing a forward perceptual bias beyond the object’s physical vanishing. This illusion does not occur with a static-noise background, suggesting that the moving object’s abrupt vanishing from the screen is usually characterized as a luminance transient, which could be considered a reset signal that terminates the predictive process and anchors the last perceived position to the physical vanishing point. In contrast, dynamic noise obscures this reset signal, leading to a positional overshoot due to incorrect predictions about the object that no longer exists. This “twinkle-goes” illusion is therefore regarded as a useful tool for investigating the dynamics of positional predictions of moving objects that are otherwise difficult to trace due to the interruptive effect of the reset signal from the offset transient in normal visual processing.

Although the phenomenology of the twinkle-goes illusion indicates that the positional prediction of a moving object can manifest as a compelling positional overshoot, little is known about its underlying mechanism. Positional predictions may rely on an object tracking mechanism that operates under limited cognitive resources (Howe and Holcombe 2012; Kwon et al. 2015a; Luu and Howe 2015). Consistent with this, the twinkle-goes illusion is reduced during simultaneous tracking of multiple moving objects (Nakayama and Holcombe 2021). Multiple-object tracking with low (~ 4–8 Hz) temporal resolution, which decreases proportionally with the number of tracked objects (Holcombe and Chen 2013; Verstraten et al. 2000), has been suggested to involve a rhythmic process that alternately allocates limited resources to the objects to be tracked (VanRullen 2016; Wutz et al. 2020). Possibly reflecting this rhythmicity, continuously moving objects appear in rhythmic motion at ~ 10 Hz and ~ 4–8 Hz when they comprise equiluminant colors (Amano et al. 2008; Arnold and Johnston 2003; Minami and Amano 2017) and second-order motions (Nakayama et al. 2018), respectively, both of which are poor stimuli for driving luminance-based motion detection systems.

By combining psychophysical and electroencephalographic (EEG) experiments, the present study tested two hypotheses regarding the twinkle-goes illusion: (1) the illusion reflects an incorrect positional prediction that overshoots the vanishing position of a moving object, and (2) such a positional prediction occurs in synchrony with neural oscillations in the aforementioned theta band. In Experiment 1, we tested the first hypothesis. If positional prediction incorrectly overshoots the vanishing position of a moving object, the illusion should be null at the time of vanishing, and should gradually increase thereafter. We measured the perceived disappearance position using a flashed probe on dynamic- or static-noise backgrounds. The illusion size was near zero with no delay and increased with probe delay, saturating ~ 120 ms after vanishing　in the dynamic condition, whereas no illusion was observed at any delay in the static condition. In Experiment 2, we tested the second hypothesis. As recent findings have suggested the existence of behavioral rhythmicity in object motion perception, the involvement of neural oscillations in the twinkle-goes illusion is worth testing. We measured the illusion size on a trial-by-trial basis by using the method of adjustment, and related its variability to EEG phase before vanishing. Such internally driven variability has frequently been used as a suitable index to examine internally driven predictions, with an inter-trial circular–linear correlation between the neural oscillation phase and continuous behavioral data utilized to examine the rhythmic nature of their relationship (e.g., Busch and VanRullen 2010; Chakravarthi and VanRullen 2012; for review, VanRullen et al. 2011). The results revealed that the illusion size correlated with the theta (3–5 Hz) phase before vanishing.

## Methods

### Experiment 1

#### Participants

Ten adults (age range: 20–33 years; four women and six men; one of whom was an author) participated in Experiment 1. All participants had normal or corrected-to-normal visual acuity and were right-handed. Except for the author, all participants were naïve to the purpose of the experiment and recruited via convenience sampling. The sample size was determined prior to data collection based on a previous study that reported the twinkle-goes illusion (Nakayama and Holcombe 2021).

All experiments were conducted in compliance with the guidelines that were in accordance with the Declaration of Helsinki (2003), and approved by the ethics committee of the Graduate School of Humanities and Sociology at the University of Tokyo. All participants provided written informed consent before participation.

## Apparatus

Visual stimuli were generated by a computer program running on MATLAB (Mathworks Inc.), Psychophysics Toolbox (Brainard 1997; Pelli 1997), and Vision Toolbox (Nakayama and Motoyoshi 2018) programming environments and presented on a gamma-corrected 22-inch cathode ray tube (CRT) screen (1280 × 960 pixels, 100 frames/s). Each pixel subtended 3.2’ at a viewing distance of 57 cm, constrained by a chin rest for binocular viewing.

## Stimuli

**Fig. 2:**
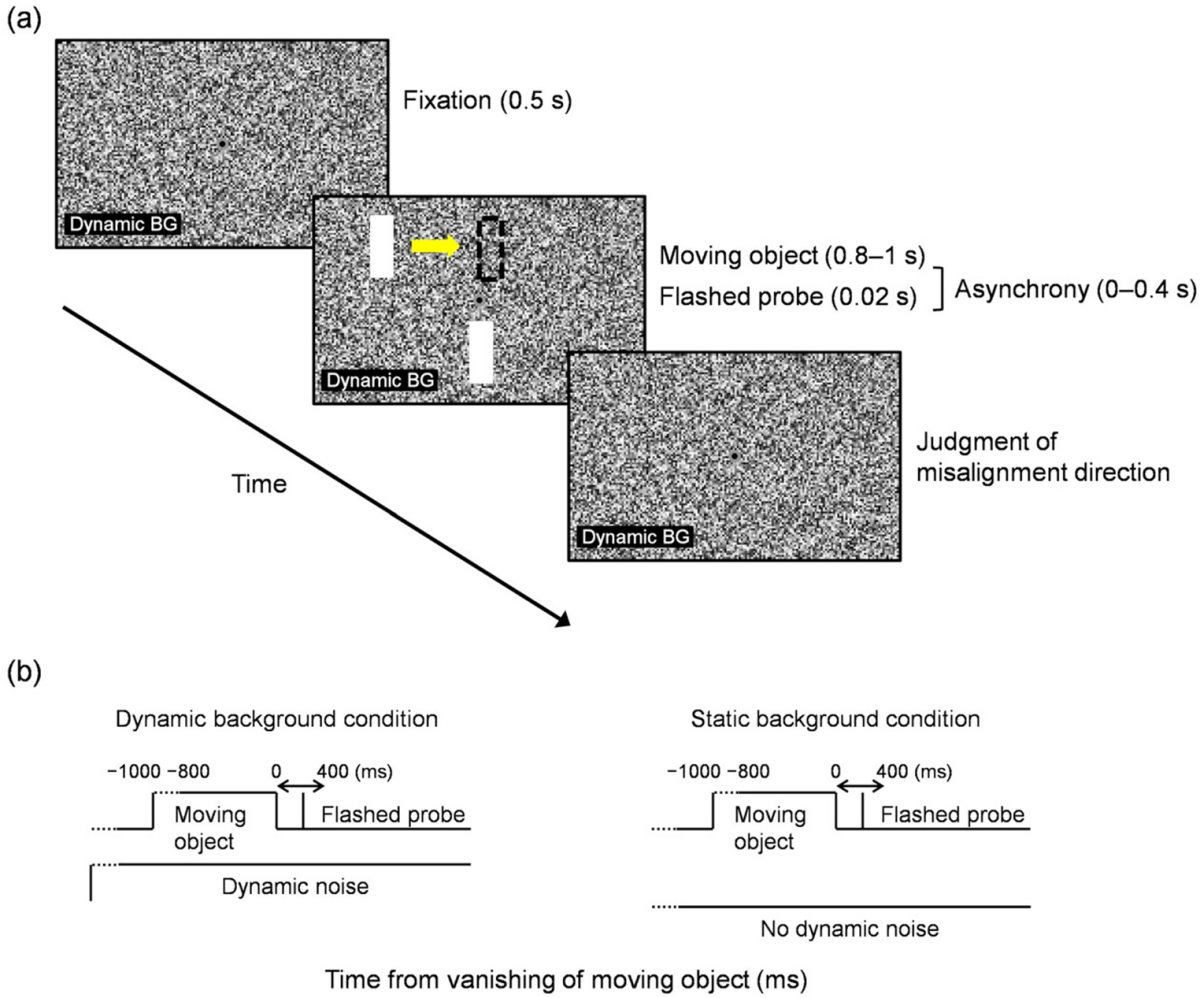
**a** Schematic of the stimulus sequence (dynamic background condition) in Experiment 1 investigating the temporal dynamics of the twinkle-goes illusion. A yellow arrow (shown here for illustrative purpose) indicates the direction of motion, while a dashed rectangle illustrates the vanishing position of the moving object, **b** time sequences for the dynamic and static background conditions, aligned relative to the vanishing of the moving object (0 ms). Dashed lines indicate trial-by-trial variations in time

The background (40.6° × 19.0°) was filled with square dots (0.13° × 0.13°) with no spacing, and luminance values randomly chosen within [0.2, 106.1] (denoting the range between 0.2 and 106.1, both inclusive) cd/m^2^. Gray and black concentric disks (0.6° outer and 0.3° inner diameters) were presented at the center as the fixation point. A vertical bar (1.0° × 4.1°, 106.1 cd/m^2^), whose center was located 3.6° above or below the horizontal meridian, moved horizontally at 19.0 °/s towards the vertical meridian (Fig. 2a). The duration (0.8–1 s) and vanishing position (± 1.0° around the vertical meridian) of the moving object were independently jittered, resulting in a variability in the starting position. As a probe, another vertical bar (1.0° × 4.1°, 106.1 cd/m^2^) was flashed for 20 ms in the vertically opposite hemifield (3.6° below or above the horizontal meridian), with a variable horizontal displacement and with a variable asynchrony relative to the vanishing time of the moving object (Fig. 2b). In the “dynamic background” condition, the background was randomly refreshed every two display frames, while in the “static background” condition, the background was filled with static noise throughout each trial.

## Procedure

In each trial, the participants viewed the stimulus display with steady fixation and reported whether the flashed probe was horizontally misaligned to the left or right relative to the vanishing position of the moving object. After each response, a new static or dynamic background with a fixation point was presented for 500 ms in the next trial. The background conditions were combined with several asynchrony conditions: 0, 40, 80, 120, 160, 200, 300, and 400 ms for the dynamic background, and 0, 80, 160, and 300 ms for the static background. Positive asynchronies denote the relative time when the probe was turned off after the moving object was turned off. For each combination of conditions, the probe displacement was adaptively modified using a staircase with a step size of 0.3° and the 1-up-1-down rule. These conditions, with 120 trials for each combination, appeared in random order. The moving object appeared in the upper or lower hemifield in random order, with its motion direction switching between leftward and rightward between consecutive trials. Consequently, the motion direction and hemifield were independent of each other.

### Analysis

The point of subjective equality (PSE) was determined as the probe’s horizontal displacement corresponding to a 50% proportion of “right” direction responses in a logistic function best-fitted with the maximum likelihood estimation. The discrimination threshold was also calculated as half the difference between the probe’s horizontal displacements corresponding to 25% and 75% proportions of “right” direction responses in the same logistic function. The goodness of fit to the logistic function was high, with an average *R*^2^ of 0.91 (*SEM* = 0.01) for the dynamic background condition and 0.90 (*SEM* = 0.03) for the static background condition. Deriving psychometric functions from staircase data enabled efficient sampling of multiple asynchrony conditions within a limited number of trials and provided reliable PSE estimates (Kingdom and Prins 2016).

For group-level statistical analyses, we performed one-sample *t*-tests and paired *t*-tests to test the null hypotheses that the illusion size (i.e., PSE) and its slope against asynchrony were zero and that the slope did not vary across background conditions, respectively. To examine the effects of background and asynchrony, we also performed repeated-measures analysis of variance (ANOVA) using Holm’s method to correct for multiple comparisons.

## Experiment 2

### Participants

Twenty adults (age range, 18–30 years; eight women and 12 men), none of whom participated in Experiment 1, participated in Experiment 2. All participants had normal or corrected-to-normal visual acuity and were right-handed. The sample size was determined prior to data collection, based on previous studies showing an inter-trial circular–linear correlation between the neural oscillation phase and behavior (Busch and VanRullen 2010; Chakravarthi and VanRullen 2012). Ethical procedures were the same as those used in Experiment 1.

### Apparatus

Visual stimuli were presented on a gamma-corrected 17.3-inch liquid crystal display (LCD) screen (1920 × 1080 pixels, 100 frames/s). Each pixel subtended 2.0’ at a viewing distance of 45 cm, constrained by a chin rest for binocular viewing.

### Stimuli

**Fig. 3:**
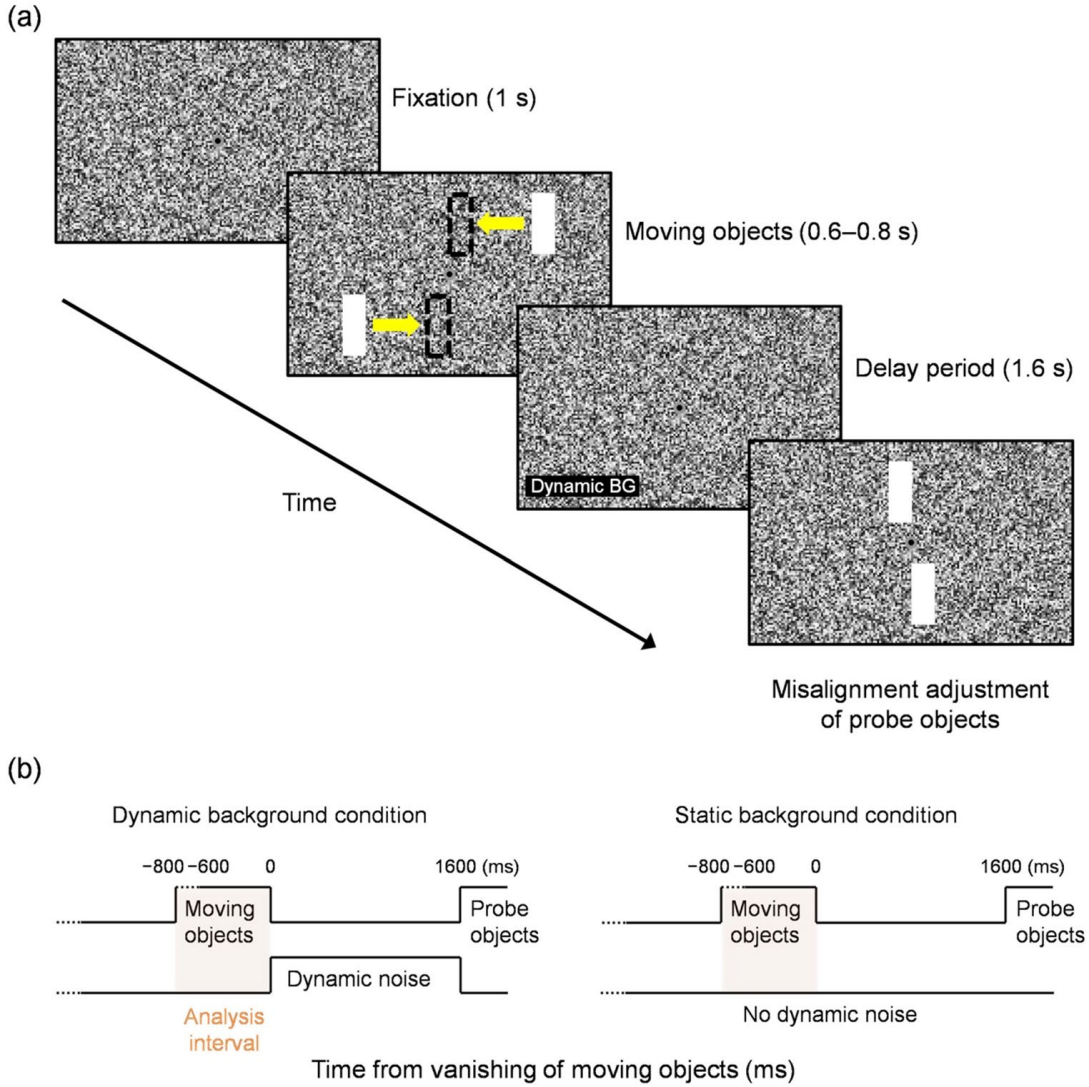
**a** Schematic of the stimulus sequence (dynamic background condition) in Experiment 2 investigating the EEG correlates of the twinkle-goes illusion. Yellow arrows (shown here for illustrative purpose) indicate the motion directions, while dashed rectangles illustrate the vanishing positions of the moving objects. (**b**) Time sequences for the dynamic and static background conditions, aligned relative to the vanishing of the moving objects (0 ms). Dashed lines indicate trial-by-trial variations in time. In each sequence, the orange box depicts the analysis interval (within [−800, 0] ms)

The background (48.4° × 18.1°) was filled with square dots (0.10° × 0.10°) with no spacing, with luminance values randomly chosen within [0.3, 117.0] cd/m^2^. Gray and black concentric disks (0.5° outer and 0.3° inner diameters) were presented at the center as the fixation point. A pair of vertical bars (1.2° × 3.3°, 117.0 cd/m^2^), whose centers were located 3.3° above and below the horizontal meridian, moved horizontally at 15.1 °/s from opposite sides towards the vertical meridian (Fig. 3a). These moving objects lasted for 0.6–0.8 s, and vanished simultaneously at horizontally symmetric positions within ± 0.2° around the vertical meridian. In both conditions, the background consisted of static noise until the vanishing of the moving objects. After vanishing, in the “dynamic background” condition, the background was randomly refreshed every two display frames for 1.6 s before reverting to static noise, whereas in the “static background” condition, the background remained static throughout the trial. As probes, another pair of vertical bars (1.2° × 3.3°, 117.0 cd/m^2^) were presented 3.3° above and below the horizontal meridian. Their presentation was deferred for 1.6 s after the vanishing of the moving objects (Fig. 3b) to prevent response-related EEG signals from being mixed with perception-related EEG signals and to allow the illusion to reach its maximum as observed in Experiment 1. The initial horizontal displacement was randomly chosen within ± 0.2° around the vertical meridian, with the pair of probes placed at horizontally symmetric positions.

According to Nakayama and Holcombe (2021), it is necessary to present dynamic noise within 80 ms after the vanishing of a moving object to ensure that the twinkle-goes illusion occurs, while increasing the noise duration within this window increases the illusion size. In the present study, both Experiments 1 and 2 met these conditions to ensure the illusion. In Experiment 1, dynamic noise began 0.5 s prior to motion onset and remained until the response (Fig. 2b). Because the dynamic noise was already present at the moment of the object’s vanishing, the post-vanishing interval fell well within the 80 ms requirement, thereby fulfilling the condition for inducing the illusion. This also prevented the dynamic noise onset from working as an undesirable masker for other visual stimuli, particularly the flashed probe. In Experiment 2, dynamic noise was started immediately after the vanishing of the moving objects (Fig. 3b), ensuring that the pre-vanishing analysis interval contained identical stimuli across conditions. In each trial, the dynamic-noise background appeared with the probability of 0.5 in random order, disabling expectations by participants.

### Procedure

In each trial, the participants viewed the stimulus display with steady fixation and adjusted the probes to match the misalignment of the disappearance positions of the moving objects using their right hand to manipulate the two buttons corresponding to the two directions of the horizontally symmetric displacements of the probes. Completion of the adjustment was indicated by pressing another button, which triggered the next trial with a refreshed static background and a fixation point of 1000 ms. The dynamic and static background conditions, with 200 trials each, appeared in random order. The directions of moving objects in the upper and lower hemifields were swapped from trial to trial.

### EEG recording and preprocessing

EEG was recorded at a sampling rate of 250 Hz using Ag–AgCl electrodes with an appropriately sized electrode cap, according to the international 10–20 system (BrainVision Recorder, actiCHamp amplifier, Brain Products GmbH; EASYCAP, EASYCAP GmbH). An additional electrode at Fpz was used as a common ground electrode. All electrodes were referenced to Cz, following common practice in EEG studies, where Cz provides a centrally located, low-impedance site that minimizes lateral bias in the recorded signals (Yao et al. 2019). The EEG was band-pass filtered (1–40 Hz) and epoched within ± 1.6 s around the vanishing of the moving objects. The EEG was decomposed into independent components using the EEGLAB runica function to identify and remove components associated with artifacts, such as eye blinks, eye movements, muscle motors, heartbeats, line noise, and channel noise (i.e., components with a brain-relatedness probability of < 0.05), using ICLabel (Pion-Tonachini et al. 2019). After the ICA-based data pruning, an average of 73.2% (*SD* = 10.5%) of the original independent components remained across the participants. This proportion of the remaining components is sufficiently large as compared with those in previous studies (e.g., 58% in Ladouce et al. 2022), and consistent with the guidelines proposed by Klug and Gramann (2021). The EEG was subsequently re-referenced offline to the average of all electrodes (recovering Cz as the average). Following the ICA-based data pruning and re-referencing, epochs exceeding ± 100 µV were excluded: in the dynamic background condition, 0 to 17 epochs were excluded for each participant (*M* = 2.4, *SD* = 4.04), and in the static background condition, 0 to 12 epochs were excluded for each participant (*M* = 1.6, *SD* = 3.00), out of a total of 200 epochs per condition. Finally, the EEG was baselined to the average potential within [− 0.4, 0] s in each epoch.

### EEG analysis

A time–frequency transform was applied to each epoch to obtain the phase angle of the EEG signals for each of the 32 electrodes at each combination of frequencies and time points using the EEGLAB newtimef function. The frequencies ranged within [3, 40] Hz in 70 steps on a log scale. Morlet wavelets were used, varying the window lengths linearly with the frequency within [1.5, 4] cycles, to reduce the time points subject to the contamination from post-vanishing activity in the pre-vanishing analysis interval. To analyze the phase dependence of the illusion, an inter-trial circular–linear correlation was computed between the phase angle and illusion size for each frequency, time point, electrode, and participant (Berens 2009) for each of the dynamic and static background conditions. These correlation values were then averaged across the participants for each of the dynamic and static background conditions.

Group-level differences between the dynamic and static background conditions in the above-described circular–linear correlations were statistically tested using cluster-based permutation tests implemented in the FieldTrip ft_freqstatistics function and its subfunctions (MonteCarlo method, 4000 iterations, maxsum criterion, alpha = 0.05 for t-tests to pre-cluster; Maris and Oostenveld 2007). As a targeted analysis to the pre-vanishing theta-band activity, clusters were first identified in the electrode domain after averaging the circular–linear correlations within specific frequency and time ranges (3–5 Hz within [− 800, 0] ms). The frequency range was determined based on prior studies that reported similar theta-band pre-stimulus correlations (e.g., Busch and VanRullen 2010; Wutz et al. 2016), while the time range corresponded to the maximum time period that contained the motion stimulus; time points later than 0 ms were excluded because the luminance transient occurring at 0 ms would evoke phase resetting in the visual response. After this initial electrode selection and averaging over the electrodes in significant clusters, the time–frequency representations were mapped out for the sake of visualization.

## Results

### Experiment 1: Temporal development of illusion size

To examine the temporal dynamics of the twinkle-goes illusion, we estimated the perceived disappearance position, defined as the probe displacement yielding equal left/right judgments (i.e., PSE), as a function of probe timing.

**Fig. 4:**
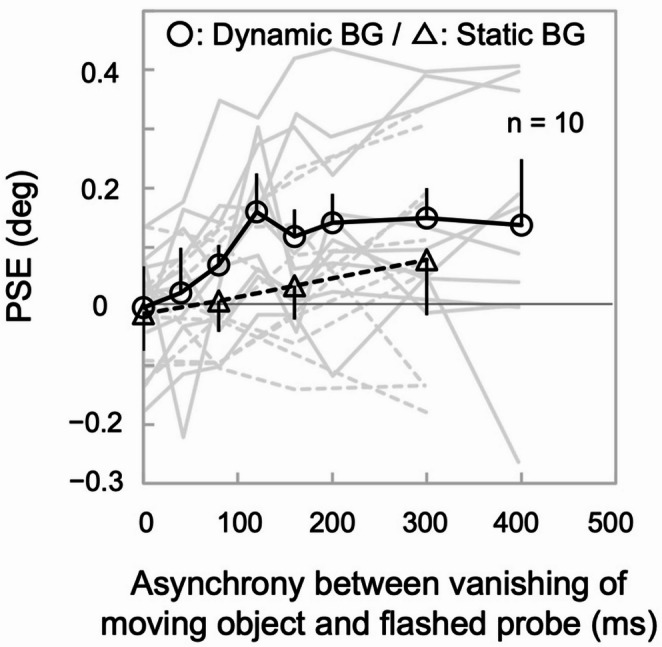
Results of Experiment 1. The abscissa shows asynchrony, defined as the relative time when the probe was turned off after the moving object vanished. The ordinate shows the PSE relative to the true vanishing position of the moving object, in degrees of visual angle, with the positive values indicating position shifts in the direction of motion. The solid and dashed curves indicate the dynamic and static background conditions, while the black and light gray curves indicate the mean and individual data, respectively. Error bars represent the Cousineau-Morey CI (Morey 2008)

In the dynamic background condition, the PSE depended on the asynchrony between the flashed probe and the vanishing of the moving bar (Fig. 4; main effect in ANOVA, *F*_(7, 63)_ = 4.41, *p* < 0.001, η_p_^2^ = 0.33). The PSE was indistinguishable from the null at the origin (*t*_(9)_ = − 0.07, *p* = 0.94, Cohen’s *d* = − 0.02 for 0 ms), and gradually increased in the direction of motion with a delay in the probe of up to 120 ms (*t*_(9)_ = 4.33, *p* = 0.002, Cohen’s *d* = 1.37 for 120 ms), after which the PSE became saturated at 0.16° on average (all *t*_(9)_ > 2.02, *p* < 0.08, Cohen’s *d* > 0.64 for 160–400 ms; see Table S1 including Bayes factors). Multiple comparisons revealed that the PSE at 120 ms (*t*_(9)_ = 3.89, *p*_holm_ = 0.007, Cohen’s *d* = 1.08), 200 ms (*t*_(9)_ = 3.45, *p*_holm_ = 0.026, Cohen’s *d* = 0.96), 300 ms (*t*_(9)_ = 3.64, *p*_holm_ = 0.015, Cohen’s *d* = 1.01), and 400 ms (*t*_(9)_ = 3.35, *p*_holm_ = 0.035, Cohen’s *d* = 0.93) were significantly different from 0 ms. The difference between 40 ms and 120 ms (*t*_(9)_ = 3.29, *p*_holm_ = 0.039, Cohen’s *d* = 0.91) was also significant. The other pairs of time points did not exhibit any significant differences (all *t*_(9)_ < 3.04, *p*_holm_ >0.08, Cohen’s *d* < 0.85; Table S1). The slope within [0, 120] ms was consistently > 0 (1.33 ˚/s [*SEM* = 0.23 ˚/s], *t*_(9)_ = 5.81, *p* < 0.001, Cohen’s *d* = 1.84), whereas the slope thereafter was not (− 0.01 ˚/s [*SEM* = 0.18 ˚/s], *t*_(9)_ = − 0.06, *p* = 0.95, Cohen’s *d* = − 0.02).

In the static background condition, the PSE did not depend on the asynchrony (main effect in ANOVA, *F*_(3, 27)_ = 1.71, *p* = 0.19, η_p_^2^ = 0.16), with the slope within [0, 300] ms not differing from 0 (0.3 ˚/s [*SEM* = 0.20 ˚/s], *t*_(9)_ = 1.55, *p* = 0.16, Cohen’s *d* = 0.49, *BF*_10_ = 0.77). Furthermore, the PSE did not significantly differ from zero at any asynchrony (all *t*_(9)_ < 1.36, *p* > 0.20, Cohen’s *d* < 0.43 within [0, 300] ms; Table S1). The lack of position shifts in the static background condition was also consistent with a previous study that showed no position shifts, regardless of asynchrony, in a similar stimulus viewed with fixation (Kerzel 2000). This may be due to the inter-individual average collapsing the opposing effects of the representational momentum or some positional overshoot at motion termination (Freyd and Finke 1984; Kanai et al. 2004), which may also involve positional prediction (Hubbard 2005), and offset repulsion (Nakajima and Sakaguchi 2016). However, the data indicated no clear bimodality in the individual differences.

For the subset of asynchronies common between the two background conditions (0, 80, 160, and 300 ms), a two-way ANOVA on PSEs, with background condition and asynchrony as factors, revealed significant main effects of background (*F*_(1, 9)_ = 10.83, *p* = 0.009, η_p_^2^ = 0.55) and asynchrony (*F*_(3, 27)_ = 4.92, *p* = 0.007, η_p_^2^ = 0.35). While their interaction was not significant (*F*_(3, 27)_ = 1.26, *p* = 0.31, η_p_^2^ = 0.12), the slope within [0, 120] ms in the dynamic background condition was larger than the slope within [0, 300] ms in the static background condition (*t*_(9)_ = 4.85, *p* < 0.001, Cohen’s *d* = 1.53, *BF*_10_ = 44.34). In addition, the pattern of statistical results was not affected even if the author’s data were excluded from the same analyses (for background, *F*_(1, 8)_ = 8.32, *p* = 0.020, η_p_^2^ = 0.51; for asynchrony, *F*_(3, 24)_ = 6.91, *p* = 0.002, η_p_^2^ = 0.46; for interaction, *F*_(3, 24)_ = 0.99, *p* = 0.41, η_p_^2^ = 0.11).

The discrimination threshold did not significantly vary by asynchrony in either background condition (main effect in ANOVA, *F*_(7, 63)_ = 0.67, *p* = 0.70, η_p_^2^ = 0.07 in the dynamic background condition; *F*_(3, 27)_ = 0.95, *p* = 0.43, η_p_^2^ = 0.10 in the static background condition). For the asynchronies common to both background conditions (0, 80, 160, and 300 ms), a two-way ANOVA on discrimination thresholds with background condition and asynchrony as factors found no significant main effects of background (*F*_(1, 9)_ = 2.59, *p* = 0.14, η_p_^2^ = 0.22), asynchrony (*F*_(3, 27)_ = 1.09, *p* = 0.37, η_p_^2^ = 0.11), or their interaction (*F*_(3, 27)_ = 0.21, *p* = 0.89, η_p_^2^ = 0.02). This confirms that the present effects in PSE were not derived from any differences in discrimination threshold.

### Experiment 2: concurrent measurements of illusion size and EEG

To examine whether neural oscillations contribute to the twinkle-goes illusion, we analyzed the relationship between trial-by-trial pre-vanishing EEG phase and perceived shift. This allowed us to test whether positional prediction is rhythmically updated in synchrony with neural activity. To allow the illusion to reach its maximum, the probe onset was delayed by 1.6 s after the moving objects’ vanishing. Consistent with the temporal dynamics observed in Experiment 1, the PSE was 0.08° (*SEM* = 0.02°) larger in the motion direction for the dynamic background condition than for the static background condition (*t*_(19)_ = 3.84, *p* = 0.001, Cohen’s *d* = 0.86).

**Fig. 5:**
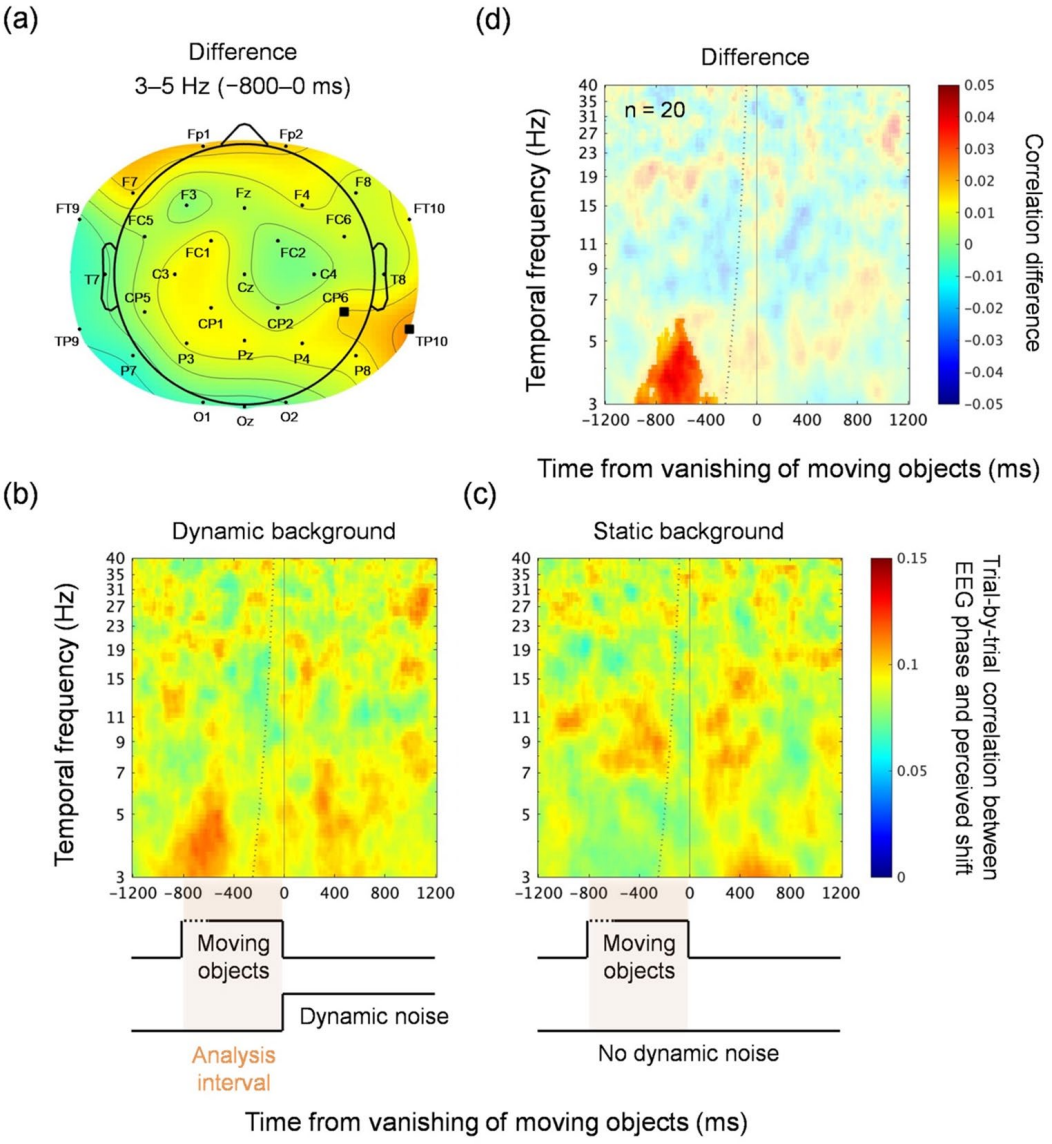
Results of Experiment 2 from the phase analyses of the EEG data. (**a**) Scalp map of the correlation difference between the dynamic and static background conditions at 3–5 Hz within [− 800, 0] ms, where the inter-trial circular–linear correlation was computed between the phase and the perceived shift. The squares indicate significant differences (cluster-corrected *p* < 0.05) identified in the permutation analysis. We selected the circular–linear correlation averaged between electrodes TP10 and CP6 to visualize the time–frequency data. The color scale is identical to that of panel (d), (**b**–**d**) time–frequency plot of the average inter-trial circular–linear correlation for the dynamic (**b**) and static (**c**) background conditions, as well as (d) their difference (b minus c; the static background condition served as a baseline for assessing the unsigned correlations observed in the dynamic background condition, helping to isolate the illusion-related component from spurious correlations due to the variability of internal noise). The saturated region of the color plot marks significant differences (cluster-corrected *p* < 0.01) identified in the permutation analysis. The dotted curve indicates the time points at which the wavelet’s time window starts to include the signals after the vanishing. In each of the time sequences illustrated at the bottom, the orange box depicts the analysis interval (within [−800, 0] ms) for the initial electrode selection

We computed the inter-trial circular–linear correlation between the EEG oscillation phase and the perceived position shift size at each of the time–frequency points, and analyzed the correlation difference between the dynamic and static background conditions. The scalp map, averaged across 3–5 Hz within [− 800, 0] ms, revealed a significant cluster over the right parietotemporal (TP10) and centroparietal (CP6) electrodes (cluster-corrected *p* = 0.016, maximum *t*_(19)_ = 3.95, maximum Cohen’ *d* = 0.88 within the cluster; Fig. 5a). For visualization, we then plotted the time–frequency representation of the circular–linear correlation averaged across TP10 and CP6 (Fig. 5b–d). Importantly, although electrodes were selected based on the 3–5 Hz activity within [− 800, 0] ms, this selection alone does not specify whether the condition difference is confined to the 3–5 Hz band or extends to other frequencies, nor whether it persists across the entire [− 800, 0] ms interval or is limited to specific time points. To address this, we formally tested condition differences across the full time–frequency plane using cluster-based permutation statistics. This analysis revealed a significant cluster selectively in the theta band (3–5 Hz) and restricted to the − 800 to − 400 ms interval (cluster-corrected *p* = 0.0003, maximum *t*_(19)_ = 5.15, maximum Cohen’s *d* = 1.15 within the cluster; Fig. 5d), indicating that the observed effect was restricted to a specific time–frequency window rather than uniformly present throughout the analysis range.

To provide a broader frequency context beyond the 3–5 Hz focus (Wutz et al. 2016), we repeated the scalp-map analysis separately for high-theta (5–8 Hz), alpha (8–13 Hz), and beta (13–30 Hz) activity within the same [− 800, 0] ms interval. This procedure allowed electrodes to be selected independently for each band, thereby testing whether condition differences might emerge at distinct scalp sites for other frequencies. However, no significant clusters were identified in these bands (all cluster-corrected *p* = 1), indicating that the illusion-related effect was specific to the 3–5 Hz range.

## Discussion

The present study psychophysically (Experiment 1) and physiologically (Experiment 2) examined the twinkle-goes illusion. Psychophysically, we found that the illusory position shift size gradually increased up to approximately 120 ms after the vanishing of the moving object (Fig. 4). Physiologically, we observed a trial-by-trial correlation between the phase angle of the 3–5-Hz oscillations before vanishing and the illusion size (Fig. 5). Taken together, these results suggest that the positional prediction of a moving object is slow-paced and rhythmically updated in synchrony with theta oscillations.

### Temporal dynamics of the illusion

Psychophysically, the illusion increased over ~ 120 ms after vanishing and then plateaued. This gradual build-up provides a distinctive signature of positional prediction: it is not instantaneous but develops within a limited temporal window. Such dynamics are inconsistent with latency-compensation models (Nijhawan 1994), which predict immediate extrapolation, and instead point to a slower updating mechanism following the loss of motion input. The overall magnitude of the shift (0.16°) was also smaller than expected from simple linear extrapolation (19 [°/s] × asynchrony [s]), suggesting that prediction is constrained by additional mechanisms such as uncertainty-based integration of position and motion signals (Kwon et al. 2015b).

### Neural oscillations and anatomical interpretation

We believe that the observed phase–shift correlation before vanishing (i.e., the phase dependence described in Fig. 5) is a proxy that reflects the correlation between the theta phase and the illusion size, which is associated with internal events only after vanishing. While the observed theta oscillations before the vanishing must remain in phase for some time after the vanishing, various reset and noise processes triggered by the vanishing event and the onset of dynamic noise render the post-vanishing correlation unintelligible. This line of reasoning shares the same idea as in previous reports, in which a correlation between phase and visual perception was observed only within the period before a visual stimulus was presented (Chakravarthi and VanRullen 2012; Mathewson et al. 2009).

The neural correlates of the illusion localized to lateral parietal–temporal electrodes (TP10, CP6), raising the possibility of right temporoparietal junction (TPJ) involvement. The TPJ has been associated with predictive processing (Geng and Vossel 2013) and theta oscillations (Seymour et al. 2018). More broadly, the parietal cortex has long been implicated in attentional tracking (Battelli et al. 2009; Culham et al. 1998, 2001) and in the accumulation of motion evidence (Shadlen and Newsome 1996, 2001), both essential for positional prediction and its overshoot (Nakayama and Holcombe 2021). The distinct right-lateralized distribution observed here may reflect task-specific demands: unlike prior studies of detection or awareness, our task required continuous estimation of position based on motion prediction, consistent with evidence implicating the right parietal cortex in temporal attentional functions (Battelli et al. 2007; VanRullen et al. 2008).

Recent studies have yielded collective evidence of the rhythmic effects of attention in synchrony with theta oscillations based on the frontoparietal cortical network (for reviews, see Fiebelkorn and Kastner 2019; Fries 2023; VanRullen 2018). Although phase dependence has been reported for attentional task performance, it has rarely been reported for visual appearance, except for the flash-lag effect (Chakravarthi and VanRullen 2012). The flash-lag effect refers to another type of mislocalization illusion, in which a briefly flashed object appears to lag behind a moving object even though they are physically aligned with each other (Nijhawan 1994). In contrast to the more anterior electrodes identified in the present study, the theta phase dependence of the flash-lag effect was identified in both occipital and frontal electrodes.

This division of labor between occipital and higher-tier areas naturally aligns with predictive coding, which posits hierarchical and reciprocal interactions between sensory inputs and top-down predictions (Rao and Ballard 1999). Occipital areas may store current sensory input, while parietal and temporoparietal regions compute predictions about upcoming positions. Such separation may prevent recursive loops between prediction and updating, and explains why non-occipital electrodes were most sensitive to phase–illusion correlations in the present study.

Although these interpretations are plausible, methodological considerations warrant caution. Finite analysis windows can contaminate pre-vanishing estimates with post-vanishing activity, but the significant effect we observed was confined to the uncontaminated window. The effect is unlikely to reflect motor preparation, as participants responded only after a long delay, and robust occipital visual evoked potentials (Fig. S1) make it improbable that posterior contributions were missed simply due to poor signal-to-noise ratio.

It remains unclear whether the observed phase dependence reflects ongoing oscillations or stimulus-driven synchronization. It is possible that the onset of motion itself evokes phase-locked theta oscillations or resets the phase of ongoing activity, thereby providing a temporal reference frame for predictive processes. Such stimulus-driven phase alignment could coordinate attentional sampling and positional prediction across extended time windows. To disentangle these possibilities, future studies will need to increase the temporal separation between motion onset and vanishing.

### A speculative model: theta-rhythmic position updating

**Fig. 6:**
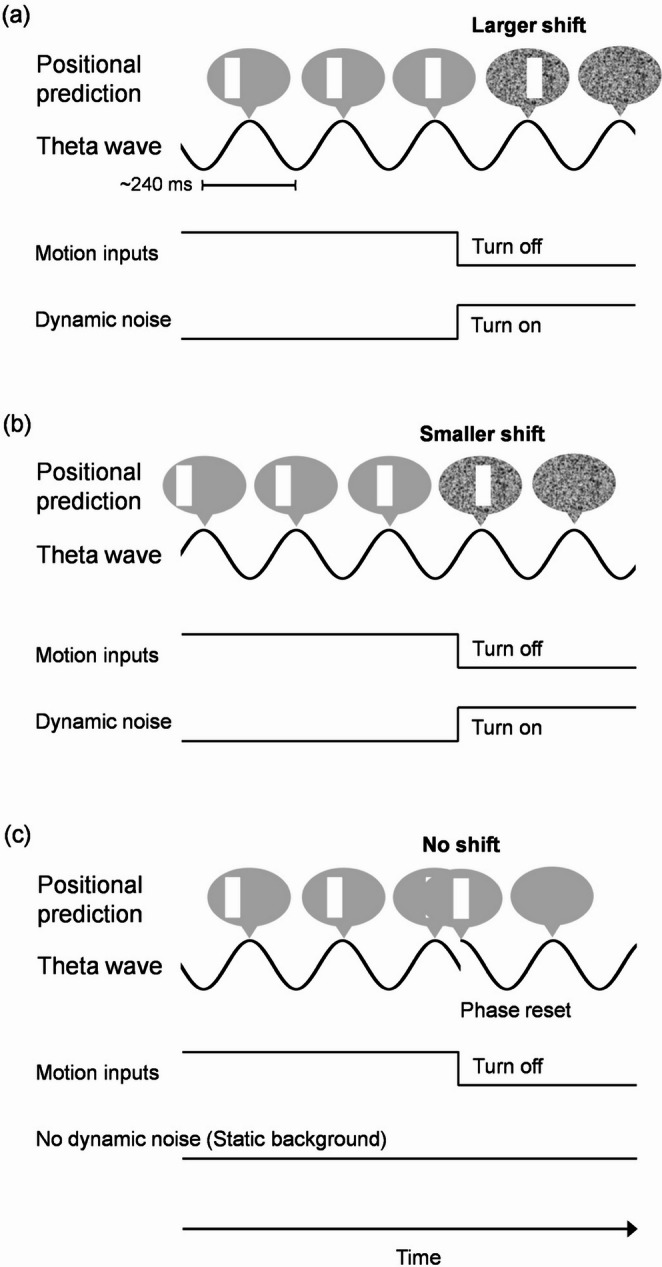
Overview of the theta-rhythmic position updating. In the dynamic background condition (**a**,** b**), the position update occurring for the first time after the cessation of motion inputs causes the latest positional prediction to overshoot the vanishing position, creating the object’s apparent position at a point predicted from the motion inputs sampled at the time of the previous update. Depending on the vanishing time relative to the theta phase, such a predictive mechanism could cause a larger (**a**) or smaller (**b**) shift in the perceived disappearance position in the first update after the vanishing, while the vanishing (based on the sensory sampling at the time of the first update) is verified in the second update. The shift size varies with the time relative to the next “peak” after the vanishing. In the static background condition (**c**), the offset transient associated with the vanishing immediately trigger the sensory sampling and reset the positional prediction to the vanishing position, producing no shift and possibly resetting the theta phase. Each rectangle represents the moving object’s horizontal position in each prediction

Integrating the psychophysical and neural findings, we propose a theta-rhythmic position updating model as a working hypothesis to be tested in future studies (Fig. 6). If position updates occur every ~ 250 ms at 4 Hz, the first post-vanishing update would fall within ~ 250 ms, averaging ~ 125 ms across trials—closely matching the psychophysical time course. Depending on the vanishing time relative to the theta phase, the first update could produce larger or smaller overshoots, while the second update confirms disappearance. Dynamic noise prevents the offset transient that normally resets prediction, allowing overshoot to manifest (Fig. 6a and b). In contrast, with a static-noise background, the offset transient resets prediction immediately, abolishing the illusion and potentially re-synchronizing theta oscillations (Fig. 6c). Although the present results are consistent with the idea of theta-rhythmic position updating, this is a speculation and the observed phase–shift correlation was modest (*r* < 0.15).

The reason we generally do not perceive any discrete motion expected from such a rhythmic position update may involve the contribution of luminance-based motion detection systems that offer dynamic signals consistent with the narrative of smoothly upfolding spatiotemporal events. This speculation is in line with previously reported phenomenology, in which continuously moving objects appear to move in a discrete fashion only when they comprise equiluminant colors (Arnold and Johnston 2003) or second-order motions (Nakayama et al. 2018), as both are poor stimuli for driving luminance-based motion detection systems. This is also consistent with case studies of akinetopsia due to lesions in motion-processing areas (Zihl et al. 1983), in which patients complain of only a discretely updated visual world.

More broadly, theta oscillations have been implicated in phase-dependent perceptual judgments (e.g., Han and VanRullen 2017) and may provide a temporal reference frame for predictive processes across domains. The relatively slow frequency of 3–5 Hz, while seemingly low for perceptual updating, may nonetheless be well suited for motor constraints such as the viscoelasticity of eye movements (saccades < 5 Hz; Martinez-Conde et al. 2004; Otero-Millan et al. 2008). Attentional, perceptual, and motor processes may thus share compatible temporal rhythms, enabling coordinated sampling and prediction across the visuomotor system.

## Supplementary Information

Supplementary Material 1

## Data Availability

All the data and code files supporting the findings of this study are available online on the Figshare public repository (https://doi.org/10.6084/m9.figshare.28831118.v1) .
