# Supplementary material for: Temporal dynamics of the twinkle-goes illusion and its relationship to neural theta oscillations
Source: Exp Brain Res. 2025 Nov 14;243(12):245. doi: 10.1007/s00221-025-07187-5 (PMC12618427; doi:10.1007/s00221-025-07187-5)
Supplement: Supplementary file 1 — Supplementary Material 1 [file 221_2025_7187_MOESM1_ESM.docx]

Supplementary Information

**Supplementary Table S1** Statistical values for PSE results. For group-level statistical analyses, in addition to the t-tests and repeated-measures ANOVA as described in the main text, we further applied Bayesian one-sample and paired *t*-tests as well as Bayesian repeated-measures ANOVA, using the JASP statistical software (JASP Team, 2019). *BF*_10_ < 0.33 is considered as substantial evidence for the null hypothesis, while *BF*_10_ > 3 is considered as substantial evidence for the alternative hypothesis.


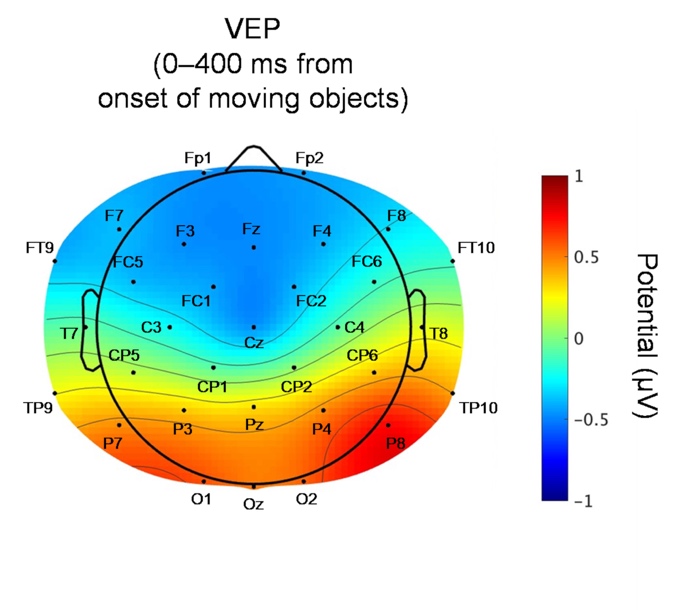


**Supplementary Fig. S1** Scalp map of visual evoked potentials averaged across participants within [0, 400] ms after the onset of the moving objects.

Supplementary references

JASP Team. (2019). JASP (Version 0.9.2) [Software].
